# Tick-Borne Viruses: Transmission and Surveillance

**DOI:** 10.3390/v18010014

**Published:** 2025-12-22

**Authors:** Anna Papa

**Affiliations:** Medical School, Aristotle University of Thessaloniki, 54124 Thessaloniki, Greece; annap@auth.gr

## 1. Brief Overview of Recent Developments

A plethora of pathogenic viruses are transmitted by ticks (tick-borne viruses, TBVs) and cause infections ranging from asymptomatic to severe cases with high fatality rates, posing a significant threat to public and animal health. Besides tick bites, TBVs can also be transmitted through direct contact with the blood or tissues of viremic patients or animals (like Crimean–Congo hemorrhagic fever virus, CCHFV) or through the consumption of unpasteurized dairy products (like tick-borne encephalitis virus, TBEV). Multiple complex factors synergistically lead to efficient virus transmission, the most critical being tick–virus–host interactions [1,2]. In addition, several drivers, including climate and ecological changes, significantly contribute to the expansion of the geographic range of TBVs, increasing the prevalence of TBV diseases [3,4]. Therefore, enhanced surveillance in ticks, animals and humans (One Health approach) is critical for the design of prevention and control measures [5,6].

Recent developments in molecular technology, including next-generation sequencing, metagenomics and other omics approaches, have increased our knowledge on virus–tick–host interactions, pathogenesis, immune response, diagnostics, vaccine research and drug discovery [7,8,9,10,11]. However, there are still gaps in the field of TBV transmission, e.g., the underlying mechanisms that are involved in the tick–virus–host interactions, the host immune response, virus adaptation to the host(s), disease severity and outcome. Similarly, data gaps exist in the mapping of TBVs’ distribution and prevalence due to a lack of integrated surveillance. This Special Issue of *Viruses* includes 11 interesting articles focusing on four major TBVs: Powassan virus (POWV, 5 articles), tick-borne encephalitis virus (TBEV, 3 articles), severe fever with thrombocytopenia syndrome virus (SFTSV, 2 articles) and CCHFV (1 article).

## 2. Articles of the Special Issue

### 2.1. Articles on Powassan Virus

POWV (family *Flaviviridae*, species *Orthoflavivirus powassanense*) is a TBV present in the United States, Canada, and Russia and causes severe neuroinvasive disease in humans. The authors of the first article (https://doi.org/10.3390/v16111653) analyzed 10 viral sequences taken from human cases, including the first confirmed lineage I infection in the United States, and showed that the sequences from humans mirror the genetic diversity seen in ticks, clustering into three major clades of the virus (lineage I, lineage II Northeast, and lineage II Midwest); since mismatches in primers/probes used in PCR assays may affect their sensitivity when applied across clades, a broad POWV PCR assay should be designed to detect the virus in clinical and tick samples [12].

The authors of the second article (https://doi.org/10.3390/v16060830) investigated whether there are strain-dependent differences in the transmission of POWV to ticks at multiple life stages, by testing the vectorial competence in five recent, low-passage POWV isolates, and showed that *Ixodes scapularis* ticks are competent vectors for all tested strains, and that the tick–virus association is stable across the various genotypes of the virus [13].

The study in the third article (https://doi.org/10.3390/v16060820) demonstrated distinct differences between POWV lineage I and lineage II regarding the clinical presentation, pathology of the central nervous system (CNS), and immune response in BALB/cJ mice when they were inoculated via the same route and dosage, suggesting different mechanisms for neuroinvasion and dissemination within the CNS [14].

The fourth article (https://doi.org/10.3390/v16020250) described the development of a Taqman-based triplex real-time PCR assay for the simultaneous and quantitative detection of POWV lineage I and lineage II in *Ixodes* ticks [15]. The assay was applied to test *I. scapularis* populations in highly endemic tick areas in Massachusetts. A comparison of the assay’s performance with a commercial Luminex panel showed that the triplex real-time PCR was more sensitive.

The aim of the last article (https://doi.org/10.3390/v16030456) was to study the vertical transmission of POWV in *I. scapularis* ticks in nature. The authors detected both RNA and infectious virus in unfed questing larvae from the field and in larvae from replete females collected from the primary tick host, suggesting that vertical transmission does occur in nature; thus, larval *I. scapularis* ticks may also present a POWV exposure risk to humans [16].

### 2.2. Articles on Tick-Borne Encephalitis Virus

TBEV (family *Flaviviridae*, species *Orthoflavivirus encephalitidis*) is endemic in Eurasia and usually causes biphasic disease in humans, presenting as febrile illness followed by CNS infection. Effective vaccines for the prevention of the disease are available.

The authors of the first article (https://doi.org/10.3390/v16091505) evaluated the VirClia IgM single-assay chemiluminescence test in 85 TBE cases in Norway and found that the specificity of the assay was 95.8%, but the sensitivity was non-inferior to the ReaScan TBE IgM rapid test (88.2% versus 94.1%) [17]. The non-reactive IgM cases were related to vaccine breakthrough infections. Therefore, isolated IgM reactive results must be interpreted with caution.

The prevalence of TBEV in 139 wild rodents trapped in endemic foci in Lithuania was estimated in the second article (https://doi.org/10.3390/v16030444); TBEV RNA was detected in 74.8% of the rodents’ brain and/or internal organ mix suspensions. It is of interest that the rate increased significantly (96.4%) following sample cultivation in Neuro-2a cells, suggesting that the procedure of the cell culture can increase the viral load to detectable levels [18]. A possible explanation for the low viral load in the majority of wild rodents might be due to the longevity of TBEV infection.

Since the interpretation of TBEV serology is hampered by a history of orthoflavivirus vaccination and by previous infections with related orthoflaviviruses, the authors of the third article (https://doi.org/10.3390/v16020286) attempted to improve TBEV IgG detection by using a combination of NS1 and EDIII antigens (instead of only NS1) using a platform for the detection of several orthoflaviviruses, including TBEV [19]. This approach potentially allows for a low-volume-based, simultaneous screening of IgG responses to a range of orthoflaviviruses with overlapping geographic distribution and clinical presentation, serving as a useful tool for patient diagnostics, vaccination responses and seroprevalence studies.

### 2.3. Articles on Severe Fever with Thrombocytopenia Syndrome Virus

SFTSV (family *Phenuiviridae*, species *Bandavirus dabieense*) is a TBV present is Asia; it causes a febrile illness often associated with thrombocytopenia and neurological symptoms. There are few reports of virus transmission from human to human, and even from cat to human. The authors of the first article (https://doi.org/10.3390/v16060874) analyzed the clinical factors associated with the course and outcome of the disease in cats (feline SFTS) and developed a scoring model to predict SFTSV infection, which contributes to the protection of cat owners, community members, and veterinarians from the risk of cat-transmitted SFTSV infection [20].

The potential mouse-to-mouse transmission of SFTSV was investigated in the second article (https://doi.org/10.3390/v16030401). It was shown that IFNAR Ab and IFNAR KO mice, as spreaders, exhibited higher transmissibility to co-housed mice than wild-type mice. Moreover, IFNAR KO mice, as recipients, were more susceptible to SFTSV infection than wild-type mice. These findings suggest that type I interferon signaling is a pivotal factor in mice intraspecies transmissibility of SFTSV in the absence of tick vectors [21].

### 2.4. Articles on Crimean–Congo Hemorrhagic Fever Virus

CCHFV (family *Nairoviridae*, species *Orthonairovirus haemorrhagiae*) is endemic in several areas of Africa, Asia and Europe and has the potential to cause a severe hemorrhagic disease in humans. It is transmitted through tick bites or direct contact with blood or tissues of viremic patients or animals.

The results of a Hungarian seroprevalence study in livestock are reported in the article on CCHFV (https://doi.org/10.3390/v16060875). CCHFV IgG antibodies were detected in 11 of 1905 (0.58%) serum samples from cattle and sheep in various districts of Hungary, suggesting that more extended surveillance and awareness among clinicians and other high-risk populations in Hungary and Central Europe are needed [22].

## 3. Future Perspectives

The complex virus life cycle in ticks and vertebrate hosts is affected by multiple factors (including tick microbiome) that play a role in transmission dynamics, level of viral load and virus dissemination into target organs. Further research studies are required to better understand the mechanisms during all steps of TBV transmission, from virus acquisition by the tick and internal replication within the tick to virus injection into a vertebrate host through the bite. This knowledge can pave the way for targeted interventions (including anti-tick vaccines) to break the infection chain early and interrupt the systematic disease. Similarly, well-designed surveillance studies will provide a better overview of the epidemiology of tick-borne viral diseases and helpful information for public health. It is expected that collaborative research networks using “One Health” approaches will generate holistic data leading to efficient control and prevention of TBVs.
